# Integrative Taxonomic Analysis Doubles Number of Species in the Central Asian Butterfly Genus *Lyela* (Lepidoptera, Nymphalidae, Satyrinae)

**DOI:** 10.3390/insects16111089

**Published:** 2025-10-24

**Authors:** Vladimir A. Lukhtanov

**Affiliations:** Department of Karyosystematics, Zoological Institute, Russian Academy of Sciences, Universitetskaya Nab. 1, 199034 Saint-Petersburg, Russia; lukhtanov@mail.ru

**Keywords:** biogeography, *Coenonympha*, Coenonymphini, *COI*, DNA barcoding, Lepidoptera, morphology, Nymphalidae, Satyrinae, taxonomy

## Abstract

*Lyela* Swinhoe, 1908 is a small Central Asian butterfly genus, in which three species were previously recognized based on comparison of wing patterns. The present study, based on an extensive population sample across the entire range of *Lyela* and using integrative taxonomy methods, confirmed the monophyly of the genus and revealed the paraphyly of the most widespread species, *Lyela myops* sensu auct. The genus is shown to include six species, *L. myops* (Staudinger, 1881) (Kazakhstan, northern Kyrgyzstan, northwestern China, and southwestern Mongolia), *L. tashkumirica* Lukhtanov, 2024, **stat. nov**. (Fergana Valley in Kyrgyzstan), *L. babatagi* Tshikolovets, 1998, **stat. nov**. (southern Uzbekistan and eastern Turkmenistan), *L. tekkensis* (Staudinger, 1886), **stat. nov**. (southwestern Turkmenistan and northeastern Iran), *L. macmahoni* Swinhoe, 1908 (Pakistan and Afghanistan), and *L. amirica* Wyatt, 1961 (Afghanistan). Each of these species represents a monophyletic unity with respect to the *COI* gene and is separated from the other species by a distinct barcoding gap and structural differences in the male genitalia.

## Simple Summary

The Central Asian genus *Lyela* has never been the subject of taxonomic revisions due to the fact that, with the exception of the widespread *L. myops myops*, the other described taxa are very local and extremely rare in entomological collections. In my research, I managed to collect material which represents all described species and the main populations of *Lyela*. Analyses of the pattern and coloration of butterfly wings, male genitalia and mitochondrial DNA barcodes show that the genus *Lyela* includes not three species, as previously thought, but six species.

## 1. Introduction

*Lyela* Swinhoe, 1908 is a small Central Asian butterfly genus known to include three species, *L. myops* (Staudinger, 1881), *L. macmahoni* Swinhoe, 1908 and *L. amirica* Wyatt, 1961 [1]. The range of the genus extends from western Kazakhstan and northeastern Iran in the west to Baluchistan (Pakistan) in the south, the Altai Mountains and Mongolia in the east, and central Kazakhstan in the north [2,3,4,5,6,7,8,9,10,11,12,13]. The species of this genus superficially resemble ringlet butterflies of the genus *Erebia* Dalman, 1816. It is therefore not surprising that the first *Lyela* species was originally described as *Erebia myops* [14], i.e., as a species of the genus currently included in the subtribe Erebiina [15]. However, already in 1908, Seitz transferred this species to the genus *Coenonympha* Hübner, 1819 [16], which is currently considered part of the subtribe Coenonymphina [15]. Taking into account the opinion of Chapman [17], who found that *Erebia myops* is distant from the true *Erebia*, Swinhoe [18] described the genus *Lyela* to include the species *L. macmahoni* (the type-species of the genus) and *L. myops*. For the same reasons, and probably unaware of Swinhoe′s work, Muschamp [19] described the genus *Dubierebia* Muschamp, 1915 (the type species: *Erebia myops*), noting that this genus occupies an intermediate position between *Erebia* and *Coenonympha*. In 1990, D’Abrera formally treated *Coenonympha*, *Lyela* and *Dubierebia* as three distinct genera, noting that *Lyela* and *Dubierebia* should possibly be regarded as congeneric with *Coenonympha* for structural reasons [20].

The closeness of *Lyela* to *Coenonympha* has been confirmed by modern molecular studies [21,22,23,24,25]. However, despite the abundance of molecular works devoted to the phylogenetic analysis of the subtribe Coenonymphina, the status of *Lyela* (genus, subgenus or synonym) and the position of this group in the system remain a subject of debate [4,25]. Even the analysis of whole genome data did not resolve this issue [26]. In the latter work, depending on the method of phylogenetic analysis, the position of the genus *Lyela* on the phylogenetic tree changes: either it appears as a sister lineage to all other Holarctic Coenonymphina, or as a sister group to *Coenonympha nolckeni* Erschoff, 1874, with the clade (*Lyela* + *C. nolckeni*) being a sister to all other *Coenonympha* species, including *Triphysa* Zeller, 1850.

The instability of the position of *Lyela* on the phylogenetic reconstructions led to the formulation of two extravagant taxonomic hypotheses. According to one of them, *Lyela* is a synonym of *Coenonympha* [21]. According to the other hypothesis, the genus *Lyela* includes the distantly similar and clearly not very closely related species *C. nolckeni* [25]. Both of the latter hypotheses are not reliably supported by molecular data and contradict the more conservative and generally accepted morphologically based concepts of *Coenonympha* and *Lyela* as two distinct genera [3,4,6,7,8,9,10,11,12]. Therefore, in my research I adhere to the traditional concept of *Lyela* as an oligotypic Central Asian genus of the subtribe Coenonymphina.

In the late 20th century and in the 21st century, several taxa of subspecific rank were described in the genus *Lyela* [27,28,29]. However, the genus *Lyela* has never been the subject of taxonomic revisions. This may be due to the fact that, with the exception of the widespread *L. myops myops*, the other described taxa are very local and extremely rare in entomological collections. In my research, I managed to collect material (including suitable for obtaining DNA barcodes), which represents all described species and the main populations of *Lyela*. Based on this material and using integrative approach, including analyses of the pattern and coloration of butterfly wings, male genitalia and mitochondrial DNA barcodes, in this article I propose a taxonomic revision of the genus *Lyela*.

## 2. Materials and Methods

### 2.1. DNA Barcoding

Standard mitochondrial DNA barcodes (658 bp fragments of *the cytochrome c oxidase subunit I* gene) were obtained for 31 samples of the genus *Lyela* at the Department of Karyosystematics of the Zoological Institute of the Russian Academy of Sciences. DNA was extracted from single legs removed from dried voucher specimens. For the majority of samples, the target 658-bp fragment of *COI* was amplified using the primers LepF1 and LepR1 [30]. For the rest of the samples, most of which were 20 or more years old, shorter overlapping fragments were amplified using the pair primer combination LepF1-MH-MR1 (311-bp amplicon) and MH-MH1-LepR1 (407-bp amplicon) followed by their concatenation [31]. Sequences were obtained using ABI 3730XL sequencer (Applied Biosystems, Waltham, MA, USA). Sequences were edited to remove ambiguous base calls and primer sequences, and assembled using SEQUENCHER (Gene Codes, Ann Arbor, MI, USA) (https://www.genecodes.com/sequencher-features, accessed on 15 September 2025). All new sequences were submitted to GenBank (accession numbers PX391132–PX391162).

### 2.2. Selection of Target Group for Phylogenetic Analysis

The phylogenetic relationships between the genera and subgenera of the subtribe Coenonymphina are not well understood [26], making it difficult to choose an outgroup for the genus *Lyela*. Therefore, I decided to include in the phylogenetic analysis, in addition to all species of the genus *Lyela*, all available species of the genus *Coenonympha*, including the phylogenetically most distant species *C. oedippus* Fabricius, 1787 and *C. nolckeni*, as well as representatives of the genera *Triphysa* and *Sinonympha* Lee, 1974. According to [24], *Oressinoma sorata* Godman & Salvin, 1868 and *O. typhla* Doubleday, 1849 were taken as the closest outgroup for all Holarctic Coenonymphina, and *Argyronympha rubianensis* Grose-Smith, 1889 was taken as a distant outgroup to root the tree.

### 2.3. DNA Matrix and Samples

A 4816 bp DNA matrix was analyzed, which was obtained by concatenating the mitochondrial *COI* gene (positions 1–1475) and five nuclear genes: *EF-1a* (positions 1476–2700), *wg* (positions 2701–3100), *RpS5* (positions 3101–3717), *GAPDH* (positions 3718–4408), and *MDH* (positions 4409–4816). This matrix was created according to the methodology of Talavera et al. [32], who proposes to combine a monolocus data set consisting of multiple DNA barcodes with a multilocus backbone data set consisting of a ‘guided’ selection of taxa with multigene sampling [32]. Matrices of this kind result in high phylogenetic resolution provided that the backbone data set includes at least one individual with multigene data for each genus and the DNA barcode set represents all or nearly all species-level taxa [32].

In the studied matrix, the backbone data set consisted of 30 specimens representing 24 *Coenonympha* species, including the most phylogenetically isolated species *C. oedippus* and *C. nolckeni*, as well as *Triphysa phryne* (Pallas, 1771), *Lyela tekkensis* (Staudinger, 1886), *Sinonympha avinoffi* (Schaus, 1927) (=*amoena* Lee, 1974, see [33] for the rationale of this synonomy), *Oressinoma sorata*, *O. typhla*, and *Argyronympha rubianensis*. In creating the backbone data set, the published nuclear and mitochondrial genes available for the subtribe Coenonymphina were used [21,22,23,24]. The DNA barcode dataset was represented by 39 *COI* fragments, 37 of which belong to the genus *Lyela* and two sequences belong to the species *C. nolckeni*.

In total, the analyzed matrix (backbone + barcode datasets) included 69 samples. Information about this matrix, including nucleotide sequences, taxon names, collection localities, field/laboratory specimen numbers, and GenBank accession numbers for the *COI* gene, is presented in full in Table S1 in Supplementary Materials. The GenBank accession numbers for the nuclear genes *EF-1a, wg, RpS5, GAPDH*, and *MDH* in this matrix are contained in publications [21,22,23,24].

### 2.4. Phylogenetic Analysis

The Bayesian analysis was performed using the program MrBayes3.2 [34]. Three schemes of the matrix partition were used: (1) analyzing the entire matrix as a single partition, (2) using partition by gene, and (3) using partition by gene for *COI*, *EF1a*, *wg*, *RpS5*, *GAPDH*, and *MDH*, and partition by codone position for *COI*. In all schemes, the Bayesian analysis of the concatenation *COI + EF1a + wg + RpS5 + GAPDH + MDH* (Table S1 in Supplementary Materials) was performed using nst = mixed, which allows MrBayes to sample all potential substitution models according to their posterior distribution. The parameter rates (gamma or invgamma) was selected separately for each partition using the program jModelTest [35]. All program settings were specified before running the program for 30,000,000 generations. The first 7500 trees (out of 30,000) were discarded prior to computing a consensus phylogeny and posterior probabilities. The consensus of the obtained trees was visualized using FigTree 1.4.4 (http://tree.bio.ed.ac.uk/software/), accessed on 15 September 202). All three partition schemes resulted in the same topology of the reconstructed consensus trees and almost identical values of posterior probabilities. Therefore, in the following section, the results are presented when the entire matrix was analyzed as a single partition.

The Maximum likelihood analysis was performed in MEGA 11 program [36] using GTR + G + I model, with 10,000 bootstrap replicates.

The *COI p*-distances (%) within and between the taxa were calculated using the MEGA 11 program [36].

### 2.5. Morphological Analysis

Morphological analysis was performed as described previously [37,38]. Briefly, to obtain preparations of male genitalia, the butterfly abdomen was placed in a hot (95 °C) 10% KOH solution for 6 min. Then, the abdomen was transferred to distilled water, and the genitalia were removed from the abdomen using a pair of dissecting needles and thin tweezers. After cleaning the genitalia from chitin residues and soft abdominal tissues, they were washed first in 45% and then in 96% ethanol and transferred to a tube with glycerol for a long-term storage. The genitalia were examined and photographed by immersing them in a container with glycerol, without pressing them with a cover glass, therefore, without distorting their native structure and.

### 2.6. Digital Documentation

Photographs of the genitalia were taken using a Leica M205C binocular microscope (Leica Microsystems, Wetzlar, Germany) equipped with a Leica DFC495 digital camera and processed using Leica Application Suite software version 4.5.0. The butterfly photographs were taken with a Nikon D810 digital camera (Nikon Corporation, Minato City, Tokyo, Japan) equipped with a Nikon AF-S Micro Nikkor 105 mm lens, using the ring full-spectrum lamp as a lighting source.

## 3. Results

### 3.1. Phylogenetic Analysis

**Figure 1:**
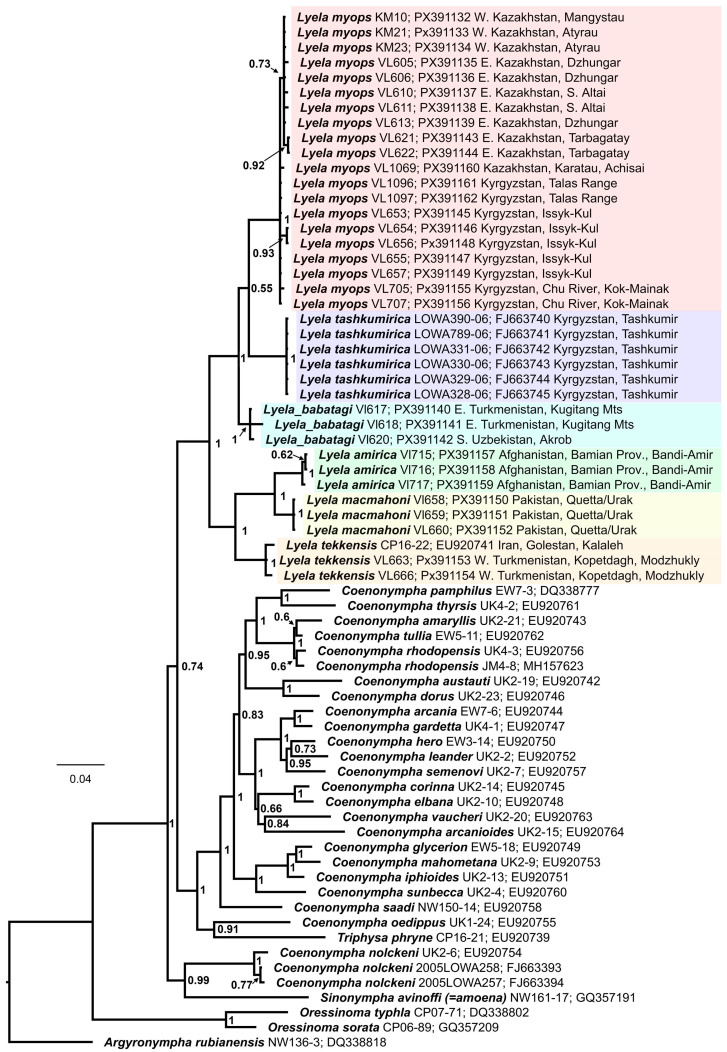
Bayesian tree (50% consensus) of *Lyela* and related genera based on analysis of the *COI + EF-1a + wg + RpS5 + GAPDH + MDH* data set. The *Lyela* species clusters are highlighted by different colours. Numbers at nodes indicate Bayesian posterior probability 0.50 and higher. Scale bar = 0.04 substitutions per position.

The Bayesian analysis revealed *Lyela* as a distinct monophyletic group (Figure 1). A sister group to *Lyela* was not identified due to low support for relationships between the *Lyela*, (*Coenonympha* + *Triphysa*) and (*C. nolckeni + Synonympha avinoffi*) clades. Two well-supported subclades were found within *Lyela*. One of them (which can be called the northern subclade) includes three well-supported clusters. These three clusters correspond to three traditionally recognized taxa known as *L. myops myops*, *L. m. tashkumirica* Lukhtanov, 2024 and *L. m. babatagi* Tshikolovets, [1998]. Since it will be argued below in the Discussion section that they represent distinct species, on the tree (Figure 1) they are designated as *L. myops*, *L. tashkumirica* and *L. babatagi*. The relationships between these three clusters were unresolved. The support for the *L. myops + L. tashkumirica* clade is extremely low (BPP = 0.55) and in fact it can be said that the three lineages of the northern subclade form a polytomy on the phylogenetic tree.

Specimens from the southern, Tien Shan part of the range of *Lyela myops myops* (Kyrgyzstan: Issyk-Kul, Chu River valley, Talas Range, Kazakhstan: Karatau Mts), differ from other samples from Kazakhstan by one fixed substitution C⇔T at position 133 of the studied DNA barcoding fragment and may probably represent a separate subspecies, the description of which will become possible in the future as more material accumulates.

DNA barcodes of the previously described subspecies *L. myops mangystavica* Lukhtanov, 1994 from western Kazakhstan were found to be identical to DNA barcodes of *L. myops myops* specimens from eastern Kazakhstan, where the taxon was originally described. This confirms the synonymization of *L. myops mangystavica* Lukhtanov, 1994 with the nominotypical subspecies [3,10].

The second subclade (which can be called the southern subclade) includes three well-supported lineages that correspond to the taxa *L. myops tekkensis*, *L. macmahoni* and *L. amirica*. Thus, the traditionally recognized species *L. myops* is revealed as a paraphyletic group, partly falling into the northern subclade (*L. myops myops* + *L. myops tashkumirica* + *L. myops babatagi*) and partly falling into the southern subclade (*L. myops tekkensis*). Within the second subclade, *L. macmahoni* and *L. amirica* are revealed as sister species, together forming a clade sister to *L. m. tekkensis*.

Maximum likelihood analysis resulted in a topology similar to that revealed by the Bayesian analysis (Figure S1). Within the genus *Lyela*, the same supported six-species clusters and the same relationships between species were identified. *Coenonympha nolckeni* was found as the sister taxon to the remaining Holarctic Coenonymphina species. A weakly supported clade (*Triphysa phryne + Synonympha avinoffi* + *C. oedippus*) was identified as the sister taxon to the remaining *Coenonympha* species. However, the relationships between these major clades outside the genus *Lyela* were low in support. Therefore, phylogenetic relationships outside the genus *Lyela* remain unclear.

MEGA 11 revealed relatively low *COI p*-distances within the studied taxa (0.00–0.79%) and relatively high *COI p*-distances between the studied taxa (2.36–6.28%) (Table 1).

### 3.2. Comparison of Male Genitalia

**Figure 2:**
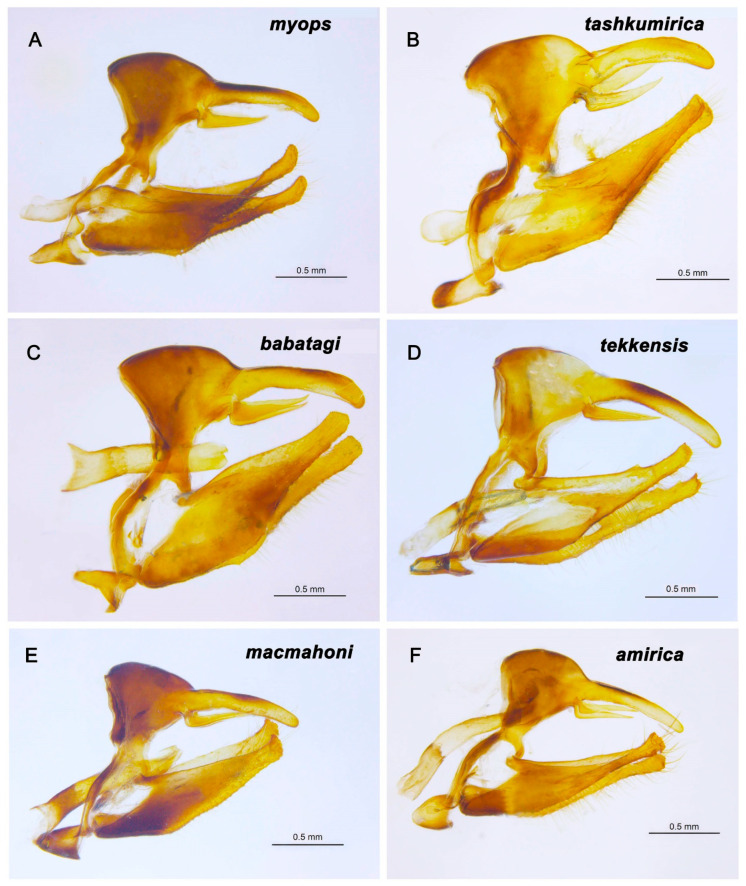
*Lyela*. Male genitalia. (**A**) *L. myops*, VL655, Kyrgyzstan, Terskey Alatau (NW edges), Karatau Mts, Orto-Tokoi, 1800–1900 m, 3 June 2005, S. Churkin. (**B**) *L. tashkumirica*, Kyrgyzstam, Jalal-Abad Rgion, near Tashkumir, 41.40° N, 72.24° E, 600 m, 4 May 1996, V.Lukhtanov. (**C**) *L. babatagi*, Turkmenistan, Kugitang Mts, near Svinzovy Rudnik, 37.80° N, 66.43° E, 800 m, 25 April 1989, V.Lukhtanov. (**D**) *L. tekkensis*, Turkmenistan, 120 km W of Ashghabat, 4–5 May 1990, V. Zolotuhin. (**E**) *L. macmahoni*, VL659, Pakistan, Baluchistan, Quetta/Urak, 2400–2700 m, 1–14 May 1983, W. Eckweiler. (**F**) *L. amirica*, Afghanistan, Bamyan prov., Bandi-Amir env., 3300 m, 1 June 2012, O. Pak.

Comparison of the male genitalia (Figure 2) revealed two groups of individuals. The first group includes individuals whose valvae have a simple structure, with the apex of the valva covered only by tiny microdenticles. Within the first group, individuals of *L. myops myops* are characterized by narrow valvae with a thinned apex. Individuals of *L. myops tashkumirica* have valvae that are wider in their middle part, without thickening in the distal part. *Lyela myops babatagi* is characterized by the presence of wide, massive valvae.

The second group includes individuals whose valvae have a complex structure, with the apex of the valva covered not only with tiny microdenticles, but also with large teeth. A probable synapomorphy of *L. macmahoni* and *L. amirica* is the expanded apical part of the valva. At the same time, the valvae of *L. macmahoni* and *L. amirica* are clearly distinguished from each other: the valvae of *L. macmahoni* are massive, and the valvae of *L. amirica* are narrow with a thinned middle and apical parts.

### 3.3. Wing Pattern

**Figure 3:**
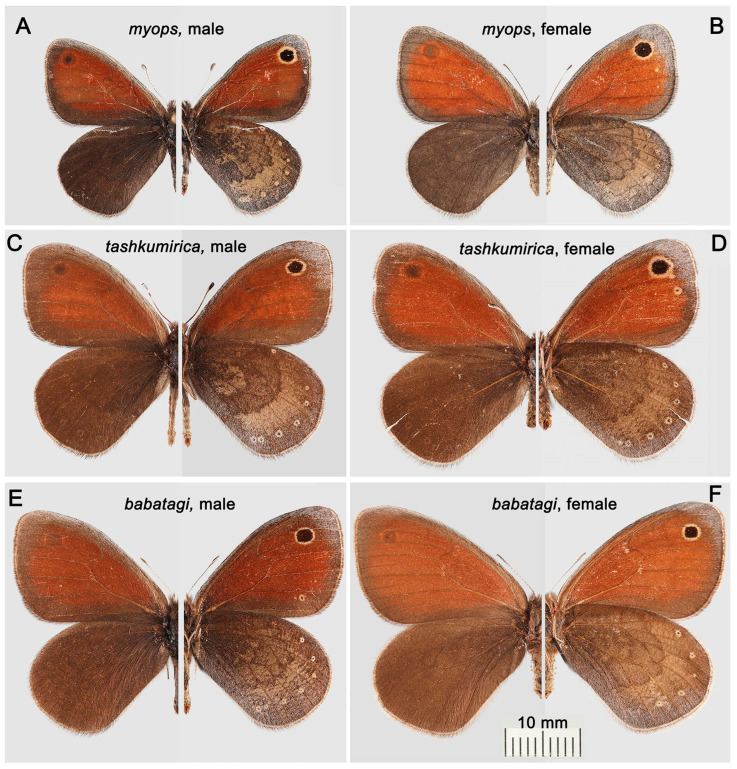
*Lyela myops*, *L. tashkumirica* and *L. babatagi*. Upperside (**left**) and underside (**right**) of the wings. The scale bar (10 mm) applies to all butterflies. (**A**) *L. myops*, male, Kazakhstan, East Kazakhstan Region, Kurchum Range (SW extreme part), Arka-Aul Mts, 48.4311° N, 83.9792° E, 490 m, 30 April 2019, V.A.Lukhtanov, in ZISP. (**B**) *L. myops*, Kazakhstan, Jetisu Region (=Taldy-Kurgan Region), Koybyn Valley, 44.21449° N, 79.50230° E, 1048 m, 30 April 2021, V.A.Lukhtanov leg., in ZISP. (**C**) *L. tashkumirica*, holotype, male, Kyrgyzstan, Jalal-Abad Region, near Tashkumyr, 41.40° N, 72.24° E, 600 m, 3 May 1996, V.Lukhtanov leg., in ZISP. (**D**) *L. tashkumirica*, paratype, female, Kyrgyzstan, Jalal-Abad Region, near Tashkumyr, 41.40° N, 72.24° E, 600 m, 3 May 1996, V.Lukhtanov leg., in ZISP. (**E**) *L. babatagi*, male, Turkmenistan, Kugitang Mts, Svinzovy Rudnik, 1400 m, 28 April 1989, V.Lukhtanov leg., in ZISP. (**F**) *L. babatagi*, female, Turkmenistan, Kugitang Mts, Svinzovy Rudnik, 1400 m, 28 April 1989, V.Lukhtanov leg., in ZISP.

**Figure 4:**
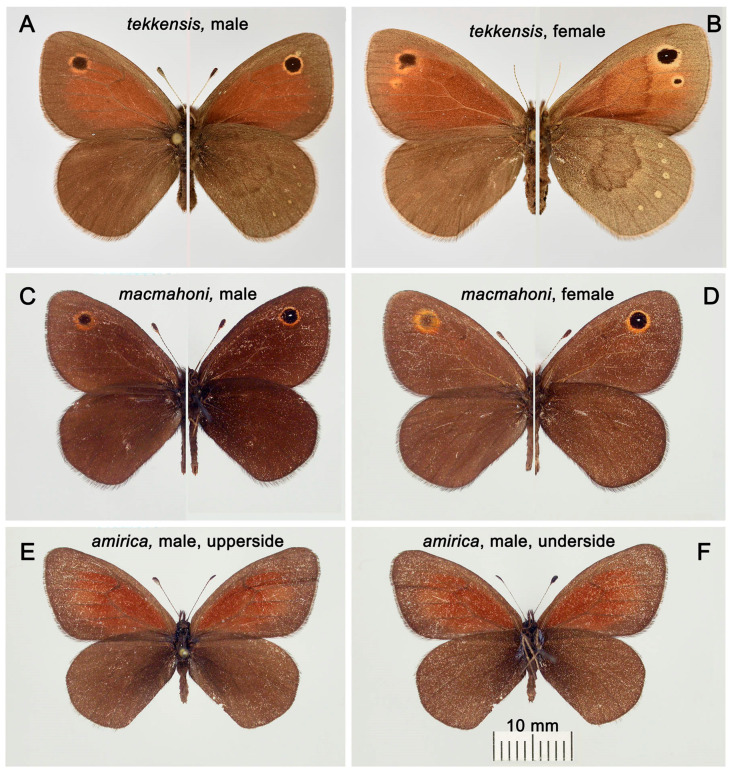
*Lyela tekkensis*, *L. macmahoni* and *L. amirica*. The scale bar (10 mm) applies to all butterflies. (**A**) *L. tekkensis*, male, sample VL665, upperside (**left**) and underside (**right**), Turkmenistan, Kopetdag, Garrygala, Parkhai, 11–20 April 1996, J.Miatleuski and A. Povilaitis, in ZISP. (**B**) *L. tekkensis*, female, sample VL666, upperside (**left**) and underside (**right**), SW Turkmenistan, Kopetdag, Modzuklu Mt, 18–21 April 1996, J.Miatleuski and A. Povilaitis, in ZISP. (**C**) *L. macmahoni*, male, upperside (**left**) and underside (**right**), [Pakistan], Baluchstan: Gwal, 31 March 1929, W.H. Evans, in NHML. (**D**) *L. macmahoni*, female, upperside (**left**) and underside (**right**), [Pakistan], Baluchstan: Gwal, 31 March 1929, W.H. Evans, in NHML. (**E**) *L. amirica*, male, upperside, Afghanistan, Koh-i-Baba Mts, Bande-i-Amir, 3000 m, 19 June 1974, S.Sakai, in NHML. (**F**) *L. amirica*, male, underside, Afghanistan, Koh-i-Baba Mts, Bande-i-Amir, 3000 m, 19 June 1974, S.Sakai, in NHML.

Butterflies of the four traditionally recognized subspecies of *L. myops* (Figure 3 and Figure 4A,B) are characterized by the presence of relatively large ocelli (eye spots) in the apical part of the forewings, as well as well-defined median bands and a number of small submarginal ocelli on the underside of the hindwings. These characters are part of the ground plan of the wing pattern of nymphalids [39] and are probably plesiomorphies. *Lyela macmahoni* (Figure 4C,D) and *L. amirica* (Figure 4E,F) are characterized by the reduction in several basic elements of the wing pattern. *Lyela macmahoni* lacks the red-brown field on the forewings and the median band on the underside of the hindwings. *Lyela amirica* lacks the apical ocellus on the forewing and the median band on the underside of the hindwings.

Among the commonly recognized subspecies of *L. myops*, *L. myops tekkensis* (Figure 4A,B) is the most differentiated with respect to wing pattern. It is markedly distinguished by the reduced size of the red-brown area on the forewings in both sexes. In *L. myops tekkensis*, this area ends near the apical ocellus. In other subspecies, this area widens toward the outer edge of the wings.

*Lyela myops tashkumirica* (Figure 3C,D) differs from *L. myops myops* (Figure 3A,B) and *L. myops mangystavica* in its noticeably larger size. In addition, in *L. m. tashkumirica*, the costal margin of the forewing is more convex, and the apex of the forewing is more rounded. In *L. m. tashkumirica*, the red-brown area on the forewings is not as wide and has a more diffuse border with the dark edge of the wing. The dark marginal stripe on the forewing is wider, and the underside of the hindwing is less contrasting, with a wider median band. *Lyela myops tashkumirica* has a noticeable external resemblance to *L. myops babatagi* (Figure 3E,F) due to the same large size, but differs in a more rounded apex of the forewing, bright whitish hairs on the costal margin of the upperside of the forewing in males, and the apical part of the underside of the forewing, which is dusty with whitish scales. In *L. myops tashkumirica*, this dusty area extends to the apical ocellus, whereas in *L. myops babatagi*, the apical ocellus is completely surrounded by the main ochre-brown background.

*Lyela myops tashkumirica* has a more contrasting underside of the hindwings with a light field between the outer edge of the median band and a row of submarginal ocelli. In *L. myops tashkumirica* this light area is covered with small grey dots. In *L. myops babatagi* this part of the wing is covered with dark strokes. In *L. myops tashkumirica* the outer edge of the median band on the underside of the hind wing has an uneven, irregular contour, and in *L. myops babatagi* this edge is wavy.

## 4. Discussion

### 4.1. Taxonomic Interpretation of the Discovered Lineages

Traditionally, the genus *Lyela* is considered to contain three species, *L. myops, L. macmahoni* and *L. amirica* [1]. If I consider the last two species of the above-mentioned, there is no doubt about their identity. *Lyela macmahoni* and *L. amirica* have striking differences in the wing pattern (Figure 4C–F). They live parapatrically in the vicinity of Kabul in Afghanistan [12], but they do not mix. They occupy different ecological niches. While *L. macmahoni* is a lower montane semi-arid species, like most other species of the genus, *L. amirica* is found in the upper montane belt [40]. As our study showed, each of them represents a monophyletic unity with respect to the *COI* gene; they are separated by a clear barcoding gap and have serious structural differences in the structure of the male genitalia. Thus, these two species have properties, which are considered essential criteria of species in virtually all species concepts [41].

The species *Lyela macmahoni* and *L. amirica* are relatively young. Taking into account previously proposed calibrations based on the mitochondrial DNA divergence in insects [42,43,44], it can be assumed that the age of their divergence is in the range of 0.65–1.5 million years. They have the lowest level of interspecific differentiation of DNA barcodes in the genus *Lyela* (2.36%), which is combined with the highest level of morphological differentiation. This is probably reflected by the fact that they live in parapatry, that is, in conditions where morphological differences can be enhanced due to selection against interspecific hybrids with reduced fertility, as is suggested for butterflies of the subgenus *Agrodiaetus* [45] and the genus *Cupido* [46].

As for the species *L. myops* in its traditional sense, when it includes all populations from Iran to Mongolia, it is characterized by a plesimorphic wing pattern type, going back to the ground plan of the nymphaloid wing pattern [39]. Our data show that it consists of four discrete clusters (*myops*, *tashkumirica*, *babatagi*, and *tekkensis*). These clusters have a level of DNA barcode differentiation exceeding that between the obvious species *L. amirica* and *L. macmahoni*. These genetic differences are correlated with differences in the structure of the male genitalia and less noticeable, but still existing, differences in wing pattern. It is also interesting that *L. myops* in its traditional sense is not monophyletic, and populations from western Turkmenistan and Iran (*tekkensis*) fall into a southern *Lyela* subclade together with *L. amirica* and *L. macmahoni*, and this subclade is contrasted by the northern subclade. Therefore, based on the criteria that were detailed earlier [47], these four clusters should be interpreted as allopatric species, *L. myops* (Staudinger, 1881), *L. tashkumirica* Lukhtanov, 2024, **stat. nov**. *L. babatagi* Tshikolovets, 1998, **stat. nov**. and *L. tekkensis* (Staudinger, 1886), **stat. nov**., and not subspecies of a single species.

### 4.2. Biogeographic Scenario

**Figure 5:**
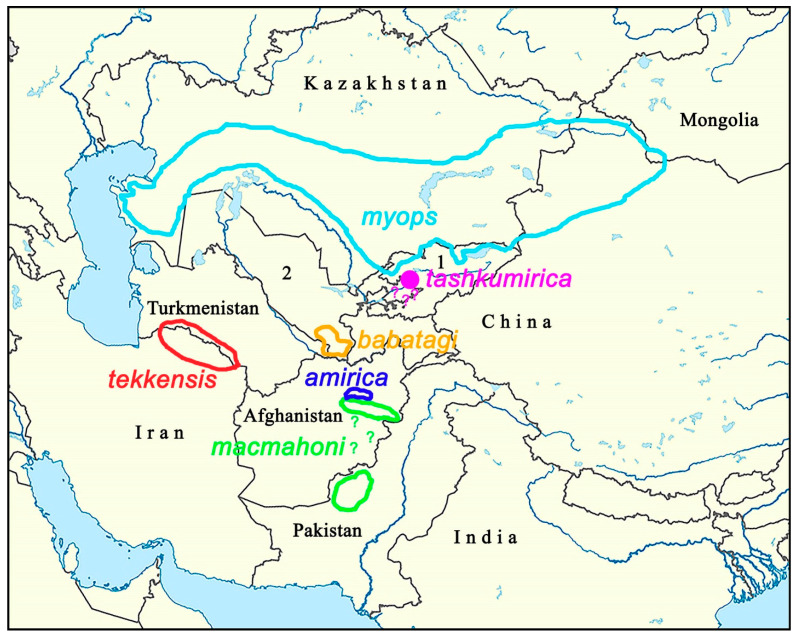
Schematic distribution map of species of the genus *Lyela*. 1 is Kyrgyzstan, 2 is Uzbekistan. A question mark (?) indicates areas where habitation is possible but not confirmed.

The conducted multilocus phylogenetic analysis, as well as previous studies [26], did not lead to a completely resolved phylogeny of the Holarctic genera and subgenera of the tribe Coenonymphina. Therefore, the sister group for the genus *Lyela* remains unknown, and this complicates phylogeographic reconstructions. Nevertheless, based on the topology of the obtained tree (Figure 1) and modern species ranges (Figure 5), it can be assumed that the origin and diversification of the genus occurred within Central Asia [25]. It can also be assumed that the first key event in the divergence of the genus was the division of the original range into two parts—northern (north and east of the Amu Darya River) and southern (south and west of the Amu Darya River). Based on the calibrations [42,43,44] and the identified level of differentiation of the *COI* gene (Table 1), this occurred 1.1–2.65 million years ago, and this dating is most likely conservative (underestimated) since it is based on the analysis of uncorrected genetic distances and does not take into account hidden nucleotide substitutions.

In the northern part of the range, the northern subclade split into three lineages. One of them is currently found in the vast semi-arid spaces of northern Central Asia (*myops*). The second is endemic to the semi-arid foothills of the Fergana basin (*tashkumirica*). The third is endemic to the semi-arid lowlands of southern Uzbekistan and eastern Turkmenistan (Kugitang Range) (*babatagi*). Species of this northern subclade are not found in either the highlands or the hyper-arid plains of southern Central Asia, providing effective geographic isolation between them. The fact that these lineages do not show even signs of a hierarchical genetic structure suggests that all three lineages originated from a common ancestor at approximately the same time (0.7–1.7 million years ago)

The species of the southern subclade form a clear hierarchical structure on the phylogeny. Based on this structure, it can be assumed that the lineage leading to *L. macmahini* and *L. amirica* arose from a common *myops*-like ancestor. This ancestor penetrated the territory of the Baluchistan Plateau and the adjacent mountains of Afghanistan, where it diverged into two species.

Thus, the genus *Lyela* is characterized by a significantly greater species diversity than previously assumed and includes six species, not three. Five of these species are local endemics with very limited ranges. This type of geographic distribution and endemism is interesting, but not unique. For example, it is found in another Central Asian butterfly genus *Phoenicurusia* Verity, 1943 (Lycaenidae) [47]. In the genus *Phoenicurusia*, *Ph. alexandra* (Püngeler, 1901) is found in southern and central Kazakhstan (analogous to *L. myops*), *Ph. dilutior* (Staudinger, 1881) is endemic to the Fergana Basin (analogous to *L. tashkumirica*), *Ph. sogdiana* (Zhdanko, 1990) is found in the lowlands of southern Uzbekistan (analogous to *L. babatagi*), *Ph. phoenicurus* (Lederer, 1870) is found in the Kopetdag in western Turkmenistan and northeastern Iran (analogous to *L. tekkensis*), and *Ph. balucha* (Howarth, 1976) is found in Pakistan and Afghanistan (analogous to *L. macmahoni*). Species of these two genera are members of the same local biota, and therefore most likely they have common history, resulting in similar species ranges.

## 5. Taxonomic Conclusions

The following taxonomic arrangement of the genus *Lyela* is proposed:

Genus *Lyela* Swinhoe, 1908 (TS: *Lyela macmahoni* Swinhoe, 1908)

Lineage *macmahoni* (=subgenus *Lyela*)

*L. macmahoni* Swinhoe, 1908 (=*macmahoni shigekoae* Sakai, 2015; =*macmahoni rekai* Sakai, 2015)

*L. amirica* Wyatt, 1961

*L. tekkensis* (Staudinger, 1886), **stat. nov.**

Lineage *myops* (=subgenus *Dubierebia* Muschamp, 1915; TS: *Erebia myops* Staudinger, 1881)

*L. myops* (Staudinger, 1881) (=*myops mangystavica* Lukhtanov, 1994)

*L. tashkumirica* Lukhtanov, 2024, **stat. nov.**

*L. babatagi* Tshikolovets, [1998], **stat. nov.**

## Figures and Tables

**Table 1:** Intra-(upper diagonal) and interspecific (all other cells) DNA-barcode *p*-distances (%) in the genus *Lyela*.

	*Myops*	*Tashkumirica*	*Babatagi*	*Tekkensis*	*Macmahoni*	*Amirica*
*myops*	0.00–0.70					
*tashkumirica*	2.89–3.97	0.00				
*babatagi*	2.52–4.27	2.75–2.91	0.15–0.61			
*tekkensis*	3.96–5.62	5.81–6.12	5.02–5.49	0.30–0.79		
*macmahoni*	4.67–6.45	6.11–6.13	5.62–5.79	4.10–4.41	0.00	
*amirica*	5.40–6.39	6.28–6.43	5.63–5.94	4.57–5.02	2.36–2.67	0.00–0.15

## Data Availability

All data supporting the findings of this study are presented in this article and the Supplementary Materials available.

## Supplementary Materials

The following supporting information can be downloaded at: https://www.mdpi.com/article/10.3390/insects16111089/s1, Table S1: List of the studied material and *COI + EF-1a + wg + RpS5 + GAPDH + MDH* matrix. Figure S1: Maximum likelihood tree of *Lyela* and related genera based on analysis of the *COI + EF-1a + wg + RpS5 + GAPDH + MDH* data set.

## Abbreviations

The following abbreviations are used in this manuscript:

BPP	Bayesian posterior probability
COI	*The cytochrome c oxidase subunit I* gene
MDPI	Multidisciplinary Digital Publishing Institute
NHML	Natural History Museum, London, UK
ZISP	Zoological Institute, St. Petersburg, Russia
