# Supplementary figures and images for: Integrative Taxonomic Analysis Doubles Number of Species in the Central Asian Butterfly Genus Lyela (Lepidoptera, Nymphalidae, Satyrinae)
Source: Insects. 2025 Oct 24;16(11):1089. doi: 10.3390/insects16111089 (PMC12653531; doi:10.3390/insects16111089)

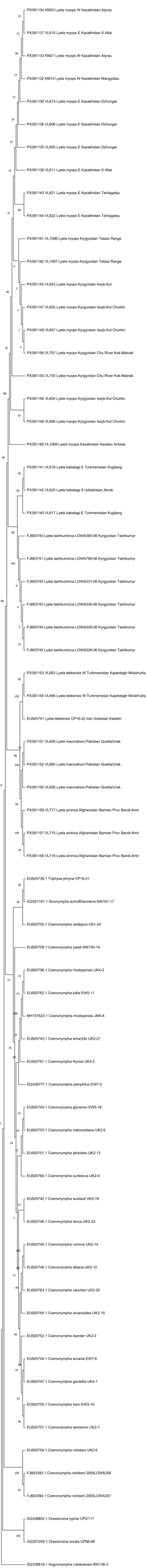

Supplement: Supplementary file 1 [file insects-16-01089-s001.zip › Figure S1. Lyela_ML_tree.pdf]
